# Can ACGME Milestones predict surgical specialty board passage: an example in Obstetrics and Gynecology

**DOI:** 10.31083/j.ceog4805168

**Published:** 2021-10-15

**Authors:** Sarah Ottum, Conrad Chao, Sejal Tamakuwala, Joshua Dean, Adib Shafi, Katherine Jennifer Kramer, Satinder Kaur, Maurice-Andre Recanati

**Affiliations:** 1 Department of Obstetrics and Gynecology, University of Cincinnati, Cincinnati, OH 45267, USA; 2 Department of Maternal-Fetal Medicine, University of New Mexico School of Medicine, Albuquerque, NM 87131, USA; 3 Department of Obstetrics and Gynecology, Emory University, Atlanta, GA 30322, USA; 4 Department of Obstetrics and Gynecology, Beaumont, Royal Oak, MI 48073, USA; 5 Department of Computer Sciences, Wayne State University, Detroit, MI 48201, USA; 6 Department of Obstetrics and Gynecology, St. Vincent’s Catholic Medical Centers, New York, NY 10011, USA; 7 Department of Obstetrics and Gynecology, Wayne State University, Detroit, MI 48201, USA; 8 Department of Obstetrics and Gynecology and NIH-Women’s Reproductive Health Research Scholar, Wayne State University, Detroit, MI 48201, USA

**Keywords:** CREOG, ABOG, Board passage, Milestones, USMLE

## Abstract

**Background:**

Multiple tools including Accreditation Council for Graduate Medical Education (ACGME) standardized milestones can be utilized to assess trainee and residency program performance. However, little is known regarding the objective validation of these tools in predicting written board passage.

**Methods:**

In this retrospective study, data was gathered on n = 45 Wayne State University Obstetrics and Gynecology program graduates over the five-year period ending July 2018. United States Medical Licensing Examination (USMLE) scores, Council on Resident Education in Obstetrics and Gynecology (CREOG) in-training scores and ACGME milestones were used to predict American Board of Obstetrics and Gynecology (ABOG) board passage success on first attempt. Significance was set at *p* < 0.05.

**Results:**

Written board passage was associated with average CREOGs (*p* = 0.01) and milestones (*p* = 0.008) while USMLE1 was not significantly associated (*p* = 0.055). USMLE1 <217 (Positive predictive value (PPV) = 96%). CREOGs <197 (PPV = 100%) and milestones <3.25 (PPV = 100%), particularly practice-based learning and systems-based practice milestones were most strongly correlated with board failure. Using a combination of these two milestones, it is possible to correctly predict board passage using our model (PPV = 86%).

**Discussion:**

This study is the first validating the utility of milestones in a surgical specialty by demonstrating their ability to predict board passage. Residents with CREOGs or milestones below thresholds are at risk for board failure and may warrant early intervention.

## 1. Introduction

The success of a residency program relies upon ensuring that residents who are in the program are learning the necessary clinical skills and academic knowledge to successfully practice and pass the specialty board examination. To ensure that residents are developing appropriately over the course of their training and developing competence appropriately, several tools have been implemented over the years.

The American medical system has evolved tremendously over the last century. The practice of medical education initially consisted of unstructured apprenticeships with virtually no requirements for medical licensure [1]. In the early 20th century William Halstead established the foundation of the American surgical residency at the Johns Hopkins School of Medicine [2]. Abraham Flexner uncovered that many training programs were of substandard quality [3], which lead to accreditation as the method of establishing uniform standards. The Accreditation Council for Graduate Medical Education (ACGME), which accredits post-graduate training programs, is tasked with “improving healthcare and population health by assessing and advancing the quality of resident physicians’ education through accreditation” [4].

Up until recently, residencies needed to show that trainees were exposed to adequate clinical volumes and a didactic program [5]. Focus then turned to producing physicians that are “competent” and possess a skill set that will “improve patient outcomes” [6, 7]. In 1998, with the support of the Robert Wood Johnson Foundation, the ACGME developed the Outcomes Project. It required physicians’ development of competencies in six broad core domains [8] (education, medical knowledge, patient care, interpersonal and communication skills, systems-based practice, and practice-based learning and professionalism), each measured by individual metrics [9, 10].

The Next Accreditation System (NAS), created out of concerns that graduates lack the readiness to practice independently [11, 12], led to a system in which resident skills are tracked through individual milestones within the six core competencies [13]. Milestones, introduced in 2014, are fine-grained developmental levels that correspond to and expand on the core competencies [14]. Numerous contributions, such as attending feedback, technical skills, clinical skills, nursing feedback, peer-review, end-of-rotation evaluations, patient feedback, and performance in simulation are used to derive individual milestone scores [15]. These 28 scores are then aggregated by an institutional clinical competency committee (CCC) and each resident’s performance is reported to the ACGME on a semiannual basis [16]. Milestone evaluations require a great deal of time and detailed effort on the part of faculty and CCC committees, but very little has been published validating the contributions of the Milestone evaluation process to quantifiable outcomes of educational efficacy. This validation is essential not only to determine the value of these processes but also to create a framework for improvements in the milestones themselves.

In parallel to the Milestones scores, the Council on Resident Education in Obstetrics and Gynecology (CREOG) has administered a multiple choice in-training examination since 1970. This test objectively assesses a resident’s cognitive knowledge in the specialty [17] while also ensuring that training programs provide adequate teaching to residents [18]. Several studies have attempted to show a correlation between CREOG scores and outcomes on the ABOG written examination [5, 19, 20]. Other studies have shown a correlation between CREOG scores and United States Medical Licensing Examination Step scores [21, 22], suggesting that these grades can be used to predict residents who will do well academically and have a high likelihood of passing written boards.

In 2016, the Obstetrics and Gynecology program at Wayne State University/Detroit Medical Center had a 50% failure rate on the American Board of Obstetrics and Gynecology (ABOG) written examination. In 2018, the failure rate was 30% in an academic program accommodating ten residents per year. These outcomes prompted this study. This study sought to determine metrics that could identify residents at risk for failure on the specialty board examination. We hypothesized that Milestone and CREOG scores would predict performance on the written Obstetrics and Gynecology (OB/GYN) board examination. To our knowledge, we are one of the few studies to date that conducted a regression analysis and built a predictive model for ABOG written board passage.

## 2. Materials and methods

Under IRB approval (042317MP2X), data for this retrospective study were collected from electronic and paper documents on residents who graduated from the Wayne State University’s Obstetrics and Gynecology Residency program between July 2013 and July 2018 and attempted to pass the ABOG written examination. Data collected during residency, and used in the analysis included the following:

### *United States Medical Licensing Examination* (*USMLE*) *Scores*:

The USMLE is a three-step examination for medical licensure in the United States and assesses a physician’s ability to apply the knowledge, concepts and principles and demonstrate patient-centered skills that constitute the basis of safe and effective care. Step 1 tests sciences basic to medical practice, Step 2 assesses clinical knowledge, while Step 3 assesses the application of medical and biomedical knowledge essential for the unsupervised practice of medicine. Scores range from 1 to 300, with mean (and standard deviation) typically 230 (19), 244 (16), 226 (15) for each step respectively [23]. USMLE step scores were analyzed individually as well as in aggregate, having defined “average USMLE score” as the average of Step 1, 2, and 3 scores.

### *CREOG scores*:

The CREOG in-training examination is a national subspecialty multiple-choice test given to all OB/GYN residents annually and offers a formative assessment of a resident’s developing medical knowledge. The overall reported standardized score are calculated with a mean of 200 and a standard deviation of 20 for all test-takers, thus 99.7% of scores range between 140–260. Internally, the raw score, which is not reported, theoretically ranges from zero to n, where n is the number of scored items on a given exam, and where n is usually around 318. CREOGs were analyzed individually and the “average CREOG” was defined as the average score obtained over the four yearly exams.

### *ACGME Milestones*:

**Fig. 1.**
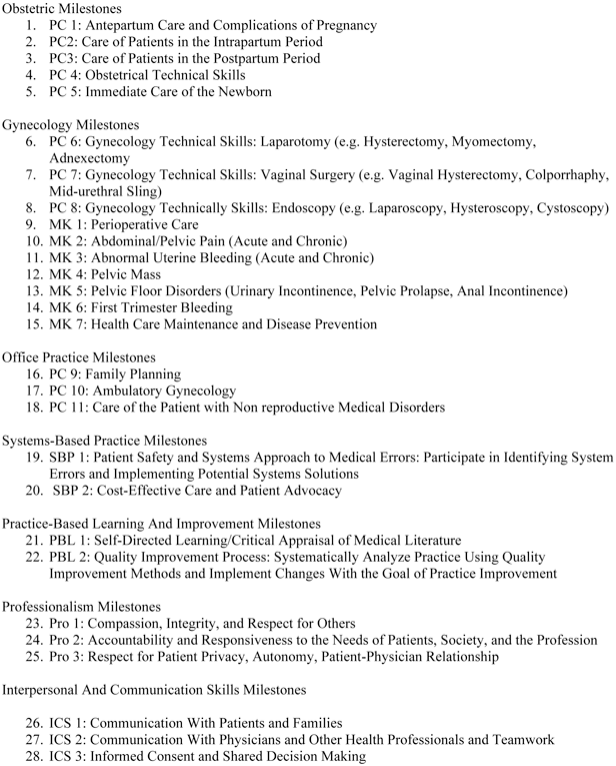
Grouping of ACGME milestone into categories. All 28 individual milestones were grouped into seven categories: obstetrics, gynecology, office, systems-based practice (SBP), practice-based learning (PBL), professionalism (PRO), and interpersonal and communication (ICS).

Milestones provide a framework for assessment of the development of residents in key dimensions of the elements of physician competency in a specialty. While many ACGME accredited specialties use Milestones, in Obstetrics and Gynecology attending-level clinicians evaluate residents with scores from 1 to 5. Level 1 corresponds to the level of an incoming intern, level 2 is below that expected at mid-residency, while level 4 is the graduation target and level 5 corresponds to an attending with some years of practice [24]. We compiled data from all 28 milestones grades from all years for all candidates, as available, at the time the study was done. To facilitate analysis, the 28 individual milestones, recorded semi-annually since the program began recording these scores, were grouped into seven categories (Fig. 1) and analyzed using matrix analysis.

### *ABOG Qualifying Exam Passage*:

Passage of the ABOG written exam was determined by the ABOG candidate status change to “active candidate”. The passing rate for first time test takers varied between 91–93%. Successful board performance was defined as passing the ABOG written boards on first attempt.

Data was de-identified and tabulated in an Excel spreadsheet and statistical analysis was performed using both ANOVA and nonparametric Mann-Whitney tests (SPSS version 26, IBM, Chicago, IL, USA). Analysis of the subgroups were performed using the F-test. Binomial logistic regression, using the criterion of *p* = 0.05 to enter and *p* = 0.01 to remove variables, was used to create a prediction equation for board passage as a binary outcome (pass or fail). Positive predictive value (PPV), negative predictive value (NPV), sensitivity and specificity were computed using standard methods.

## 3. Results

### 3.1 Descriptive statistics on cohort of residents

The dataset, which includes residents who were enrolled from July 2013 to 2018, is comprised of 45 residents, 16 males (35.5%) and 29 females (64.5%). Out of 45 graduates, 9 failed their ABOG written boards on first attempt.

### 3.2 Predictor of board passage

**Fig. 2.**
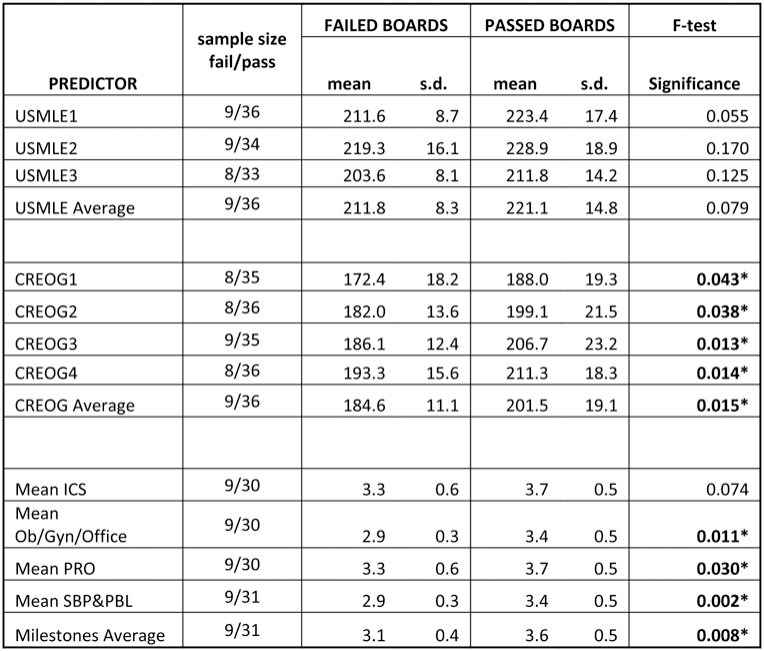
Predictors of board passage. The cohort of residents who passed ABOG written exam on first attempt was compared with those that failed the exam. Mean scores and standard deviation for each predictor variable are shown, along with *p*-values (* denotes *p* < 0.05).

**Fig. 3.**
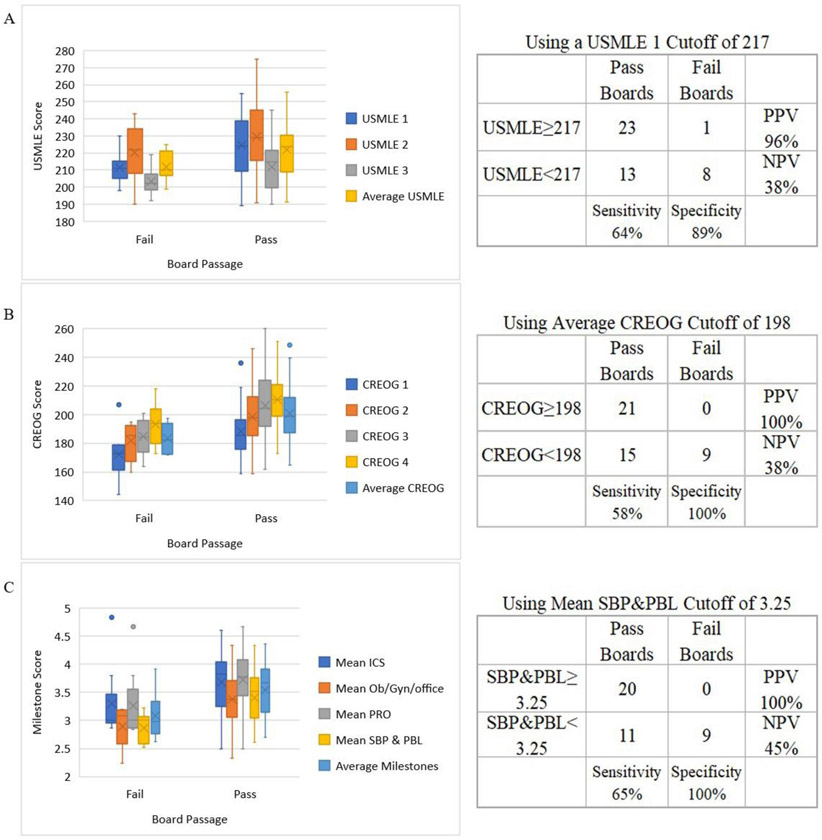
Predictors of board passage. The cohort of residents who passed ABOG written exam on first attempt (n = 36) was compared with those that failed the exam (n = 9). **Left:** Distribution showing mean (X), median (line), first & third quartile and range are depicted graphically, dots represent outliers. **Right:** Prediction accuracy. Contingency tables at specified cutoff values for pass and fail groups. Panels include (A) USMLE scores, (B) CREOGs, and (C) Milestones.

We looked for predictors of successful board performance and compared metrics from passing and failing groups (Fig. 2). Average USMLE score were not significantly associated with board passage (at *p* < 0.05), however USMLE1 came closest (*p* = 0.055). Using a cutoff of ≥217 on USMLE1, we can predict 8 of the 9 board failures and 21 of the 36 passes (PPV = 95.8%, NPV = 38%, Sensitivity = 63.9%, Specificity = 88.9%) (Fig. 3A).

Academic knowledge, such as average CREOG score, is associated with board passage (*p* = 0.015). Using a cutoff of ≥198 we are able to predict all failures and 21 out of the 36 passes (PPV = 100%, NPV = 37.5%, Sensitivity = 58.3%, Specificity = 100%) (Fig. 3B).

Clinical metrics, such as mean milestones were also significantly correlated with board passage (*p* = 0.0085). In particular, the average SBP (*p* = 0.01) and PBL (*p* = 0.002) milestone score were most strongly associated with board passage. Combining both SBP and PBL together (SBP&PBL) provided the highest association (*p* = 0.002) of any single or combination of metrics. Using a cutoff for the average SBP&PBL of ≥3.25 we are able to predict all fails and 20 of 31 passes (PPV = 100%, NPV = 45%, Sensitivity = 64.5%, Specificity = 100%) with 73% accuracy (Fig. 3C). Board passage was also associated with other milestones including office-based practice (*p* = 0.01) and professionalism (*p* = 0.03) (not shown). Rate of improvement, year-to-year in milestones was not correlated with board passage.

**Fig. 4.**
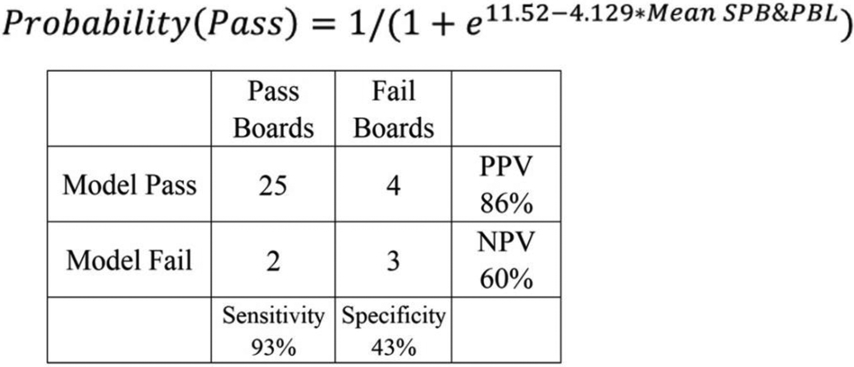
Binomial Logistic Regression Model. Mean SBP&PBL can be used to calculate the probability of passing. Insert depicts 2 × 2 contingency table for the model.

A stepwise binomial logistic regression confirms that SBP & PBL are the best predictors of board passage and were the only metrics which entered the regression. The prediction equation has a Nagelkerke R^2^ = 0.425 (*p* = 0.014) and is 82% correct in predicting pass/fail (PPV = 86%, NPV = 60%, Sensitivity = 92.6%, Specificity = 42.9%) (Fig. 4).

## 4. Discussion

The shared academic responsibility between a residency program and a resident is critical for the combined and individual success of each entity. Early markers identifying residents at risk of failure on the written examination or having trouble acquiring the clinical skills necessary for independent practice are essential to enable program directors to identify and assist struggling residents.

Milestones were the best predictor of passing the ABOG written exam. Milestones measure clinical knowledge of a topic as a resident progress over five levels, from uncomplicated management, to more complicated cases, and ultimately to a level expected for independent practice [25]. While Milestone scores are somewhat subjective, at our institution, a clinical competency committee, attended by numerous attendings from various subspecialties as well as private (non-faculty) physicians offer differing perspectives and a balanced consensus. This approach helps regulate and moderate grades assigned by faculty members who are uncomfortable giving low scores [26] or who are influenced by personal biases or opinions of residents [27]. Often, milestone scores from the CCC committee were very much in-line with resident self-assessment [15, 16] and were as objective as possible in measuring clinical performance. The bulletin for the ABOG basic written examination [28] suggests that the exam spans mostly clinical topics in obstetrics, gynecology and office practice including topics on postoperative care, patient evaluation, surgical management and possible complications. It is likely that one of the reasons that Milestones most closely predict a resident’s performance on this exam is because the focus of the exam is clinical and mirrors the practical experience as well as practical knowledge needed to take care of patients. As residents progress through the steepest part of the learning curve over the first year, significant gains in clinical knowledge are made that are reflected in improved milestone scores [29]. The rate of milestone improvement, however, did not correlate with board passage. This may be because different residents progress at different rates; however the absolute score is more reflective of performance.

Our data suggests that two milestones were most significant. PBL reflects self-directed learning and improvements that allow a doctor to become a more masterful clinician. This is a measure of internal motivation, desire to improve, acceptance of responsibility for one’s own learning combined with the initiative and self-discipline to complete goal-oriented self-directed learning. Individuals who score high in this area are typically able to identify their own deficiencies and limits in knowledge, analyze their own practice, incorporate feedback from faculty and assimilate evidence from scientific publications into their practice [30]. These life-long basic study skills translate into residents who enjoy learning, as well as teaching their junior colleagues, and typically have the knowledge and self-confidence to do well clinically as well as on board exams, as demonstrated in our results. The other highly predictive Milestone, SBP, reflects on a physician’s awareness of (and responsiveness to) the healthcare universe in which they deliver care. Clinicians who score high generally exhibit strong interpersonal and communications skills and professionalism enabling them to interface well with other members of the team. They emphasize shared-decision-making, advocate for quality patient care, actively seek to improve the quality of care and find creative solutions to limitations inherent to the system [31]. Importantly, they take steps to enhance patient safety while embracing personal responsibility and quality improvement. While the ABOG qualifying exam is a written exam, patient safety is one of the important areas tested and this foundational Milestone touches on all cross-content areas examined, including ethics, communication and health literacy [32]. This may help explain the importance of this Milestone in our results.

Performance on the ABOG written examination was correlated with CREOG and Milestone scores. Our analysis showed that average CREOGs greater than 198 were associated with board passage. This finding was similar to other studies which determined that a score of 200 either as a PGY-4 or two times throughout residency correlated with a 100% probability of passage [19], or a score over 202 on the third year CREOG was predictive of board passage [20]. While other studies found that CREOGs were not useful in identifying residents at risk of board failure [5], our analysis validated the utility of Milestones. Milestones were more predictive than other metrics such as CREOGs. One possible explanation is that the ABOG exam essentially tests a candidate’s ability to interpret clinical data and derive a treatment plan [28], while CREOG questions tend to test knowledge differently. Prior publications have demonstrated that in-training exam scores are expected to increase with each year of training as more knowledge is accumulated [18, 33]. In our study, year-to-year improvement on CREOGs did not significantly correlate with board passage but score value did [17]. Unlike other studies which found that USMLE Step 1 correlated with board passage and that Step 1 >200 was suggestive of successful outcomes on boards [21], our study did not echo these findings. In our cohort, Step 1 was not significantly correlated to board passage at a *p* < 0.05 and mean Step 1 scores in those who failed written boards was 211. This may be due to factors specific to our program or to our propensity to highly rank medical students with high USMLEs.

One potential weakness in this study is the unusually high failure rate in our program during the study period, compared to the nationwide failure rate of 3–9%. Possible confounding factors, which may affect the applicability of our study, may include particulars of our residency program at the time, such as service to teaching balance, structure of the curriculum and composition of our medical residents. However, data collected from our single site offers consistency in faculty evaluators (the same individual faculty members) and clinical rotations (no changes in the number of weeks on the various rotations), making the residents one of the only variables. Without a large number of individuals failing, it would be statistically difficult to characterize the unique characteristics of residents who have failed. The limitation, however, is the smaller sample size. Strengths of this study include the predictive regression analysis. Because of the diversity in our program, including residents who transferred in from other programs, our results may not be applicable to other institutions.

## 5. Conclusions

We have validated the utility of the OB/GYN residency Milestones by demonstrating their ability to predict OB/GYN written board passage, a critical accomplishment that constitutes an objective external assessment of educational efficacy and attainment. The encouraging results from this pilot study suggest that a larger study encompassing Milestone data from all residency programs across all surgical specialties would confirm these preliminary findings.

## Data Availability

The datasets that support the findings of this study are available from the corresponding author (MAR) upon reasonable written request.
